# Auger electron-emitting EGFR-targeted and non-targeted [^197^Hg]Hg-gold nanoparticles for treatment of glioblastoma multiforme (GBM)

**DOI:** 10.1186/s41181-025-00367-2

**Published:** 2025-07-17

**Authors:** Madeline K. Brown, Zhongli Cai, Constantine J. Georgiou, Shaohuang Chen, Yumeela Ganga-Sah, Valery Radchenko, James T. Rutka, Raymond M. Reilly

**Affiliations:** 1 Department of Pharmaceutical Sciences, Leslie Dan Faculty of Pharmacy, University of Toronto, Toronto, ON M5S 3M2 Canada; 2 Life Sciences Division, TRIUMF, Vancouver, BC Canada; 3 Department of Chemistry, Simon Fraser University, Vancouver, BC Canada; 4 Department of Chemistry, University of British Columbia, Vancouver, BC Canada; 5 Division of Neurosurgery, The Hospital for Sick Children, Toronto, ON Canada; 6 Division of Neurosurgery, Department of Surgery, Temerty Faculty of Medicine, University of Toronto, Toronto, ON Canada; 7 Department of Medical Imaging, University of Toronto, Toronto, ON Canada; 8 Princess Margaret Cancer Centre, University Health Network, Toronto, ON Canada

**Keywords:** Glioblastoma multiforme (GBM), Gold nanoparticles, ^197^Hg, Auger electrons, Panitumumab, Epidermal growth factor receptors (EGFR), Convection-enhanced delivery

## Abstract

**Background:**

We describe here radiation nanomedicines for glioblastoma multiforme (GBM) composed of gold nanoparticles (AuNPs) that integrate the Auger electron-emitter, ^197^Hg. [^197^Hg]Hg-AuNPs were conjugated to anti-epidermal growth factor receptor (EGFR) panitumumab or were non-targeted. Our aim was to compare the cytotoxicity and DNA-damaging properties in vitro of panitumumab-[^197^Hg]Hg-AuNPs and non-targeted [^197^Hg]Hg-AuNPs on U251-Luc human GBM cells and estimate their cellular dosimetry. We further aimed to compare the biodistribution in vivo of panitumumab-[^197^Hg]Hg-AuNPs and [^197^Hg]Hg-AuNPs after convection-enhanced delivery (CED) in NRG mice with U251-Luc tumours in the brain and estimate the absorbed doses in the tumour and surrounding margins of healthy brain.

**Results:**

[^197^Hg]Hg-AuNPs (26.8 ± 6.4 nm) were produced with a radiochemical yield of 98 ± 1% by incorporating ^197^Hg into the Turkevich synthesis method, forming a mercury-gold amalgam. Panitumumab-[^197^Hg]Hg-AuNPs exhibited high affinity (K_D_ = 1.8 × 10^–9^ mol/L) binding to EGFR-positive U251-Luc cells. The binding of panitumumab-[^197^Hg]Hg-AuNPs to U251-Luc cells was 15-fold higher than [^197^Hg]Hg-AuNPs, and internalization and nuclear uptake were 12-fold and 18-fold greater, respectively. Panitumumab-[^197^Hg]Hg-AuNPs caused 84-fold more DNA double-strand breaks (DSBs) in U251-Luc cells than [^197^Hg]Hg-AuNPs. Panitumumab-[^197^Hg]Hg-AuNPs were ninefold more effective at reducing the clonogenic survival of U251-Luc cells than [^197^Hg]Hg-AuNPs. Panitumumab-[^197^Hg]Hg-AuNPs were twofold more cytotoxic than non-radioactive panitumumab-AuNPs (*P* = 0.04) and fivefold more cytotoxic than panitumumab (*P* = 0.01). The absorbed doses in the nucleus of U251-Luc cells treated in vitro with panitumumab-[^197^Hg]Hg-AuNPs or [^197^Hg]Hg-AuNPs were 8.8 ± 2.9 Gy and 0.6 ± 0.1 Gy, respectively. SPECT/CT imaging showed that panitumumab-[^197^Hg]Hg-AuNPs and [^197^Hg]Hg-AuNPs were strongly retained at the infusion site in the brain after CED up to 7 d in NRG mice with orthotopic U251-Luc tumours. Uptake of panitumumab-[^197^Hg]Hg-AuNPs in the tumour-bearing right hemisphere [484.5% injected dose/g (%ID/g)] was 172-fold and 579-fold greater than in the healthy left hemisphere and cerebellum, respectively. The uptake of [^197^Hg]Hg-AuNPs (423.9% ID/g) in the tumour-bearing right hemisphere was 85-fold and 64-fold higher than the left hemisphere and cerebellum, respectively. Most normal tissue uptake was < 1% ID/g, except for kidneys (9–20% ID/g), spleen (3.5–6.6% ID/g) and liver (0.6–3.3% ID/g). Dosimetry showed that 58% of the tumour received > 190 Gy for CED of 1.0 MBq of panitumumab-[^197^Hg]Hg-AuNPs vs. 0.6% of the tumour for non-targeted [^197^Hg]Hg-AuNPs, but both agents deposited > 50 Gy in 95% of the tumour. Doses decreased dramatically to 1.7 and 3.3 Gy at 1–3 mm from the tumour edge for panitumumab-[^197^Hg]Hg-AuNPs and [^197^Hg]Hg-AuNPs, respectively.

**Conclusion:**

Radiation nanomedicines incorporating the AE-emitter, ^197^Hg administered by CED are a promising approach to treatment of GBM. Panitumumab-[^197^Hg]Hg-AuNPs are particularly attractive due to their EGFR-mediated binding, internalization and nuclear importation in GBM cells, which amplifies their in vitro cytotoxicity.

**Supplementary Information:**

The online version contains supplementary material available at 10.1186/s41181-025-00367-2.

## Introduction

Glioblastoma multiforme (GBM) is the most common and lethal primary brain tumour in adults (Lau et al. 2014; Wilson et al. 2014). The standard treatment for patients diagnosed with GBM involves maximal surgical resection followed by concurrent X-radiation and temozolomide (TMZ) chemotherapy then adjuvant TMZ chemotherapy (Stupp et al. 2005). Despite an aggressive treatment regimen, the median survival of patients with GBM is < 2 years (Wilson et al. 2014). This is due to the infiltrative nature of GBM which makes complete tumour resection difficult, resulting in recurrence that often arises within 2 cm of the surgical margins (Wallner et al. 1989). Recurrent GBM tends to be more aggressive and treatment resistant, leading to death in almost all patients (Vaz-Salgado et al. 2023). There is no accepted standard-of-care for patients with recurrent GBM (van Linde et al. 2017). Strategies to eradicate residual disease at the surgical margins could improve patient outcomes by slowing or preventing recurrence. We envision that a radiation nanomedicine could be infused via convection enhanced delivery (CED) into the surgical cavity to treat residual tumour. Radiation nanomedicines consist of nanoparticles complexed to radionuclides that emit α- or β-particles or Auger electrons (AE) (Reilly et al. 2024). CED employs catheters that are precisely inserted in the brain to slowly infuse therapeutic agents under a pressure gradient that optimizes local distribution. CED has been previously studied for delivery of a range of therapeutic agents to GBM (Kreatsoulas et al. 2024). For example, β-particle-emitting ^186^Re-labeled liposomes were safely administered by CED for radiation treatment of recurrent GBM in a recent Phase 1 clinical trial (ReSPECT-LM; ClinicalTrials.gov ID: NCT05034497) (Brenner et al., 2023). ^186^Re-liposomes administered by CED extended the median survival to 12 months vs. 9 months historical survival for recurrent GBM. CED offers advantages compared to intravenous administration for treating GBM since it circumvents the blood–brain-barrier (BBB) which poses a major obstacle to delivery of effective therapeutic doses to tumours in the brain. CED further deposits these agents in close proximity to the tumours while restricting normal tissue uptake and toxicity since the BBB poses a barrier to redistribution outside the brain (D'Amico et al. 2021).

We previously reported that CED of a radiation nanomedicine composed of gold nanoparticles (AuNPs) complexed to the β-particle emitter, ^177^Lu through a metal-chelating polymer (MCP) (1.1 MBq; [^177^Lu]Lu-MCP-AuNPs) was highly effective for treating luciferase-transfected U251-Luc human GBM tumours in the brain in NRG mice via CED (Georgiou et al. 2023), demonstrating prolonged survival > 150 d in 5/8 mice treated with [^177^Lu]Lu-MCP-AuNPs vs. 39 d for normal saline-treated mice and 45 d for mice treated with non-radioactive AuNPs. Comparable results were obtained in immunocompetent C57BL/6J mice with murine GL261 glioma tumours treated by CED of [^177^Lu]Lu-MCP-AuNPs (2.7 MBq) combined with anti-programmed cell death-1 (PD1) checkpoint immunotherapy (Georgiou et al. 2024).

An alternative to β-particles for treating GBM that has not been explored are AE. Although AE have low energy (< 25 keV), it is deposited over an ultrashort distance (< 1 µm), yielding high linear energy transfer (LET = 4–23 keV/µm) up to 230-fold greater than β-particles (0.1–1 keV/µm), making AE potent for killing cancer cells (Ku et al. 2019). Moreover, high LET radiation such as AE may directly cause lethal DNA double-strand breaks (DSBs) in cancer cells, while β-particles rely on indirect DNA damage mediated by reactive oxygen species (ROS), which is susceptible to tumour hypoxia (Wenker et al. 2025). Mercury-197 (^197^^g^Hg; t_1/2_ = 64 h) is an attractive AE-emitter due to its abundant AE emissions (23 AE/decay) and low frequency of *γ*- or X-ray emissions, which minimizes off-target irradiation of normal cells (Ku et al. 2019). We describe here the construction of a novel AE-emitting radiation nanomedicine for treating GBM composed of AuNPs that integrate ^197^Hg by forming a stable Hg-Au amalgam. To maximize the potency of the AE emitted by ^197^Hg, we further conjugated these [^197^Hg]Hg-AuNPs to the anti-epidermal growth factor receptor (EGFR) monoclonal antibody, panitumumab (Vectibix®, Amgen) which binds and internalizes them into GBM cells. EGFR are overexpressed on 50–60% of GBM (Heimberger et al. 2005) and a nuclear localizing sequence (NLS) in the EGFR routes internalized ligands including antibodies to the cell nucleus, where AEs are most damaging to DNA and lethal (Lo et al. 2006). The cytotoxicity and DNA-damaging properties of panitumumab-[^197^Hg]Hg-AuNPs and non-targeted [^197^Hg]Hg-AuNPs in U251-Luc human GBM cells were compared. Further, the tumour and normal tissue biodistribution of panitumumab-[^197^Hg]Hg-AuNPs and [^197^Hg]Hg-AuNPs after CED in NRG mice with U251-Luc tumours in the brain were compared and dose estimates for the tumour and surrounding margins of healthy brain tissue calculated.

## Methods

### Cell culture and EGFR characterization of U251-Luc cells

Luciferase-transfected U251-Luc human GBM cells were provided by Dr. James Rutka (Hospital for Sick Children, Toronto, ON, Canada). U251-Luc cells were cultured at 37 °C/5% CO_2_ in DMEM supplemented with 10% FBS (Gibco-Invitrogen, ThermoFisher, Ottawa, ON, Canada). The EGFR expression of U251-Luc cells was compared to MDA-MB-231 cells (1.5 × 10^5^ EGFR/cell) and MDA-MB-468 human breast cancer cells (1.3 × 10^6^ EGFR/cell) (Reilly et al., 2000) and U87MG human GBM cells which have low EGFR expression (Zhu et al., 2013) by flow cytometry [Supplementary Information (SI) Additional file 1: Fig. S1]. Based on this analysis, the estimated EGFR expression of U251-Luc cells was 2.3 × 10^5^ receptors/cell.

### Synthesis and characterization of [^197^Hg]Hg-AuNPs

^197m^Hg/^197^Hg were co-produced at TRIUMF (University of British Columbia, Vancouver, BC, Canada) from a natural gold target (Additional file 1: Fig. S2a) by the ^197^Au(p,n)^197m^Hg/^197g^Hg reaction. Due to the longer half-life of ^197g^Hg (64.1 h) compared to ^197m^Hg (23.8 h; Additional file 1: Fig. S2b), the predominant form at the time of synthesis of [^197^Hg]Hg-AuNPs (> 48 h post-production) was ^197g^Hg. Hereafter, we use ^197^Hg to describe ^197m^Hg/^197g^Hg. [^197^Hg]Hg-AuNPs were synthesized by incorporating [^197^Hg]HgCl_2_ into the synthesis of AuNPs to form a mercury-gold amalgam, based on the Turkevich method, which relies on trisodium citrate reduction of aq. tetrachloroauric acid (HAuCl_4_) (Kimling et al., 2006). Briefly, 100 µL of 0.25 mM HAuCl_4_ (> 99.9%, Sigma-Aldrich) was mixed with 4–5 MBq (50 µL) of [^197^Hg]HgCl_2_ in 40 mL of autoclaved 18.2 MQ water in a 250 mL round-bottom glass flask. After 20 min of heating at 100 °C, 400 µL of 500 mM trisodium citrate (> 99%, Sigma-Aldrich, St Louis, MO, USA) was added. All glassware was thoroughly cleaned prior to use by coating the inner surface for 2.5 h with 20 mL aqua regia consisting of 3 parts of concentrated HCl (Sigma-Aldrich) and 1 part of concentrated HNO_3_ (WardScience, Mississauga, ON, Canada) then rinsing with 18.2 MQ water. The pH was 4.0 after addition of [^197^Hg]HgCl_2_ measured by pH Test Strips (VWR Chemicals BDH, Mississauga, ON, Canada). The solution was refluxed at 100 °C for 10 min. Formation of AuNPs was recognized by the appearance of a red colour. The solution was cooled for 30 min to room temperature (RT) and transferred into 50 mL conical tubes (Corning Incorporated, Corning, NJ, USA). The efficiency of incorporation of ^197^Hg was measured by ultracentrifugation of a 1.0 mL sample at 15,000 × g for 15 min and separating free ^197^Hg (supernatant) from [^197^Hg]Hg-AuNPs (pellet). The supernatant and pellet were measured in a radioisotope dose calibrator (CRC-15R, Capintec Inc, Ramsey, NJ). [^197^Hg]Hg-AuNPs and non-radiolabeled AuNPs were characterized for size, shape and stability in medium with 10% fetal bovine serum (FBS), phosphate-buffered saline (PBS) or artificial cerebrospinal fluid (CSF) as described in the SI.

### Conjugation of panitumumab to [^197^Hg]Hg-AuNPs

To target EGFR overexpressed on U251-Luc human GBM cells, [^197^Hg]Hg-AuNPs were conjugated to panitumumab (Vectbix®, Amgen, Mississauga, ON, Canada). Briefly, 1 × 10^11^ AuNPs in 1 mL of ddH_2_O in a 1.5 mL Eppendorf tube were incubated with a tenfold excess of panitumumab (1 × 10^12^ molecules; 0.3 µg) pre-mixed at room temperature (RT) for 30 min with 0.006% polyoxyethylene (20) sorbitan monolaurate (Tween-20, Sigma Aldrich) to stabilize the AuNPs against aggregation (Shih et al. 2014). This mixture was vortexed and incubated for 30 min at RT on a shaker (Brinkman Orbimix 1010) at 170 rpm. The reaction ratio of panitumumab/[^197^Hg]Hg-AuNPs was selected based on studies in which 1 mL of AuNPs (1 × 10^11^ AuNPs) were incubated with a 10- to 250-fold excess of panitumumab mixed with [^111^In]In-DOTA-panitumumab (2.4 MBq; 333.2 µg) to radiotrace and quantify the conjugation of panitumumab to AuNPs [see Additional file 1]. [^111^In]In-DOTA-panitumumab was synthesized as previously reported (Facca et al., 2022) and described briefly in the SI. AuNPs conjugated to [^111^In]In-DOTA-panitumumab under these conditions were evaluated for binding and internalization into U251-Luc human GBM cells by cell fractionation studies (Additional file 1: Fig. S3) to further select the optimal conjugation conditions. Panitumumab binds to AuNPs via a gold-thiol bond formed by a cysteine residue in panitumumab as reported for conjugation of epidermal growth factor (EGF) to AuNPs (Song et al., 2017). At completion of the reaction between [^197^Hg]Hg-AuNPs and panitumumab + Tween 20, 10 µg of lipoic acid-polyethylene glycol 2 kDa (PEG_2K_-LA) (Nanocs, New York, NY, USA) was added to PEGylate the AuNPs to increase their stability and prevent aggregation. The mixture was vortexed and incubated for 15 min at RT, then centrifuged at 10,000 × g for 15 min. The pellet was isolated and resuspended in 0.1 µg/mL of PEG_2K_-LA in ddH_2_O to maintain the PEGylation and stability of the AuNPs and centrifuged at 10,000 × g for 15 min. This process was repeated twice, to isolate PEGylated panitumumab-[^197^Hg]Hg-AuNPs.

The binding of panitumumab-[^197^Hg]Hg-AuNPs to EGFR on U251-Luc cells was determined in a saturation radioligand binding assay. Briefly, 1 × 10^5^ U251-Luc cells in 1.5 mL Eppendorf tubes were incubated for 3 h at 4 °C with increasing concentrations (0.01–22.1 nmoles/L) of panitumumab-[^197^Hg]Hg-AuNPs in PBS to measure total binding (TB) or were pre-incubated with an excess of panitumumab (27.7 µmoles/L) for 2 h to block EGFR prior to incubation with panitumumab-[^197^Hg]Hg-AuNPs to measure non-specific binding (NSB). Specific binding (SB) was calculated by subtracting NSB from TB. Cells with bound panitumumab-[^197^Hg]Hg-AuNPs were centrifuged at 400 × g and the supernatant removed. The cell pellet was washed once with PBS and centrifuged at 400 × g and the supernatant removed. The cell pellet was then measured in a γ-counter (PerkinElmer Model 1480, Waltham, MA, USA). TB, NSB and SB were plotted vs. the concentration of panitumumab-[^197^Hg]Hg-AuNPs. The curves were fitted to a one-site receptor-binding model by Prism® Ver. 10.3.1 software (GraphPad, San Diego, CA, USA) to estimate the dissociation constant (K_D_) and maximum binding sites per cell (B_max_).

### Cell binding and internalization studies

The binding and internalization of panitumumab-[^197^Hg]Hg-AuNPs and [^197^Hg]Hg-AuNPs to U251-Luc cells was measured (Facca et al., 2022). Briefly, 5 × 10^4^ U251-Luc cells were seeded into wells in a 24-well plate (Sarstedt, Nümbrecht, Germany) containing 400 uL of DMEM + 10% FBS medium and cultured at 37 °C/5% CO_2_ overnight. The medium was removed and panitumumab-[^197^Hg]Hg-AuNPs or non-targeted [^197^Hg]Hg-AuNPs (1 × 10^11^ AuNPs; 0.26 MBq) in 200 µL of DMEM + 10% FBS were added to the wells and incubated for 19 h at 37 °C/5% CO_2_. The medium was removed and collected. The cells were then rinsed twice with 250 µL cold phosphate-buffered saline, pH 7.4 (PBS; Gibco, Fisher Scientific) and placed on ice. Cell surface-bound activity was displaced and collected by incubating the cells with 250 µL of 0.2 M sodium acetate and 0.5 M NaCl at pH 2.5 for 10 min on ice, then rinsing the cells twice with 250 µL of cold PBS. The cells were then lysed by incubation with 250 µL of Nuclei EZ Lysis buffer (Sigma-Aldrich) overnight at 4 °C. The cells were detached with a cell scraper (Sarstedt) and transferred to 1.5 mL Eppendorf tubes. The tubes were centrifuged at 500 × g (Eppendorf Model 5424 centrifuge, Hamburg, Germany) for 5 min to separate the nucleus activity in the pellet from cytoplasm activity in the supernatant. This process was repeated twice, and nuclei were rinsed with 250 µL of cold PBS. The activity remaining in the medium or bound to the cell surface, or in the cytoplasm and nucleus fractions were measured in a γ-counter (PerkinElmer Model 1480). The percentage of activity in each fraction was calculated and compared for panitumumab-[^197^Hg]Hg-AuNPs and non-targeted [^197^Hg]Hg-AuNPs. EGFR mediated binding and internalization of panitumumab-AuNPs by GBM cells was further assessed by comparing the uptake by U251-Luc cells with high EGFR expression or U87-MG human GBM cells with low EGFR (Additional file 1: Fig. S1) and GL261 murine glioblastoma cells with negligible EGFR (Guo et al. 2019). Panitumumab does not bind to murine EGFR (Tabrizi et al. 2006), thus GL261 cells are further non-immunoreactive for human EGFR. These studies were performed using [^111^In]In-DOTA-panitumumab-AuNPs as described in the SI.

### Clonogenic survival and γ-H2AX assays and cellular dosimetry

The effect of exposure of U251-Luc cells to panitumumab-[^197^Hg]Hg-AuNPs or non-targeted [^197^Hg]Hg-AuNPs on their clonogenic survival was determined. Briefly, 5 × 10^4^ U251-Luc cells were seeded into wells containing 400 µL of DMEM in a 24-well plate (Sarstedt) and cultured overnight at 37 °C/5% CO_2_. The medium was removed and the cells rinsed with 200 µL PBS. Then 0.17 or 0.26 MBq (1.3 MBq/mL) of panitumumab-[^197^Hg]Hg-AuNPs or non-targeted [^197^Hg]Hg-AuNPs (1 × 10^11^ AuNPs) in 200 µL of DMEM + 10% FBS were added to the wells. Control wells were incubated with an equivalent amount of unlabeled panitumumab-conjugated AuNPs or unconjugated AuNPs or an equivalent amount of panitumumab (based on the number of panitumumab conjugated to [^197^Hg]Hg-AuNPs) or were left untreated. The cells were incubated at 37 °C/5% CO_2_ for 24 h. Then 700–1,400 cells were seeded into 6-well plates (Sarstedt) containing 3 mL of fresh DMEM with 10% FBS and cultured for 8 d at 37 °C/5% CO_2_. The medium was removed, and the surviving colonies stained with 1% methylene blue in a 1:1 mixture of ethanol and water. The plates were rinsed with water and imaged on a ChemiDoc gel imaging system (BioRad, Mississauga, ON, Canada) and colonies (> 50 cells) were counted automatically using ImageJ software (U.S. National Institutes of Health, Bethesda, MD, USA) and a customized macro (Cai et al., 2011). The plating efficiency (PE) was calculated by dividing the number of surviving colonies by the number of cells seeded. The clonogenic survival fraction (SF) was calculated by dividing the PE for treated cells by that of untreated cells. Based on the results of cell fractionation studies, the absorbed doses in the nucleus of U251-Luc cells incubated with 0.26 MBq (1 × 10^11^ AuNPs) in the clonogenic survival assays were estimated for panitumumab-[^197^Hg]Hg-AuNPs and [^197^Hg]Hg-AuNPs as described in the SI and using previously reported methods (Cai et al. 2023, 2017).

DSBs in U251-Luc cells caused by AE emitted by panitumumab-[^197^Hg]Hg-AuNPs or non-targeted [^197^Hg]Hg-AuNPs were measured by immunofluorescence microscopy probing for phosphorylated histone-2AX (γ-H2AX) as previously reported (Cai et al., 2009). Briefly, U251-Luc cells treated as described earlier in the clonogenic survival assays were seeded onto glass coverslips (Ted Pella, Redding, CA, USA) placed into wells containing 500 µL of DMEM in a 24-well plate (Sarstedt) and cultured for 24 h at 37 °C/5% CO_2_. The cells were then fixed with 2% paraformaldehyde + 0.5% Triton 100, permeabilized by 0.5% Igepal CA630 (Nonidet P-40) and non-specific binding blocked with 2% BSA in PBS and 1% donkey serum (Sigma-Aldrich) for 1 h before probing with anti-phospho-histone H2AX (Ser139, clone JBW301) mouse IgG_1_ (Millipore-Sigma, Oakville, ON, Canada). Cells were then incubated with Alexa Fluor 488 donkey anti-mouse IgG (H + L) (Invitrogen Molecular Probes, Carlsbad, CA, USA). Finally, the coverslips containing immunostained cells were mounted on microscope slides using Vectashield® Mounting Medium containing 4',6-diamidino-2-phenylindole (DAPI) to counterstain the nucleus. Images of γ-H2AX foci in the nucleus were acquired with the Zeiss Axio Observer.Z1/7—Apotome 2 microscope fitted with a Plan-Apochromat 63 × /1.40 Oil DIC M27 objective and laser scanning mode set as plane with two solid state laser lines (353 and 488 nm) chosen on the acquisition software Zen Blue. At least 50 cells were imaged per slide. The light source intensity, exposure time, and image resolution (0.072 μm/pixel) were kept constant for all image acquisitions. Images were processed to calculate the mean integrated density of γ-H2AX foci per unit nucleus area vs. untreated controls using ImageJ and a customized macro.

### SPECT/CT imaging and biodistribution studies

Orthotopic U251-Luc tumors were stereotaxically established in the right cerebral hemisphere of NOD-Rag1^null^IL2rγ^null^ (NRG) mice as previously reported (Georgiou et al. 2023). After two weeks, tumours were confirmed by bioluminescence imaging (BLI). Groups of 4–5 tumour-bearing NRG mice were then stereotoaxically infused by CED of 5 µL of panitumumab-[^197^Hg]Hg-AuNPs or non-targeted [^197^Hg]Hg-AuNPs (0.7 MBq; 7 × 10^11^ AuNPs) at a flow rate of 0.5 µL/min as previously reported (Georgiou et al. 2023). Whole body single photon emission computed tomography/computed tomography (SPECT/CT) images were obtained at 1 h, 1 d, 3 d and 7 d post-infusion (p.i.). For SPECT/CT, mice were anaesthetized with 2% isoflurane in O_2_ and imaged in the prone position on a trimodality (SPECT/CT/PET) system (NanoScan®, Mediso, Budapest, Hungary) equipped with 4 NaI(Tl) detectors fitted with 0.85 mm multi-pinhole collimators. Images were acquired with an energy window centered (± 10%) around the 77 keV γ-photopeak of ^197^Hg. CT images were acquired with 50 kVp X-rays, 980 μA, and a 300 ms exposure time, isotropic voxel size of 125 μm and maximum field-of-view with 1:4 binning. Images were reconstructed using TeraTomo 3D Normal Dynamic Range Monte Carlo-based reconstruction protocol with a 128 × 128 reconstruction matrix, with three subsets of data undergoing 48 iterations applied with CT-based attenuation and scatter correction. SPECT and CT images were co-registered by InterView Fusion software (Ver. 3.09; Mediso). Following the final images obtained at 7 d p.i., the mice were sacrificed and the uptake of activity [percent injected dose/g (%ID/g)] in the brain and other tissues was measured in a γ-counter (PerkinElmer). The brain was segmented into the right hemisphere (tumour-bearing), healthy left hemisphere and cerebellum (3 segments). The brain was also examined visually ex vivo for deposition of AuNPs recognized by a darker red colour.

### Estimation of radiation absorbed doses and dose mapping

To understand the potential therapeutic effects of panitumumab-[^197^Hg]Hg-AuNPs vs. non-targeted [^197^Hg]Hg-AuNPs, the absorbed dose distribution around the infusion point in the brain for CED of 1.0 MBq was generated based on SPECT/CT images obtained from 1 h to 7 d p.i., respectively. A volume of interest (VOI) encircling the mouse head in the SPECT/CT images was drawn to measure the maximum voxel value (MBq/mL) and the sum of activity (MBq) at 1h, 1, 2, 3, 4 and 7 d p.i. to calculate the conversion factor from the voxel value to Bq for ^197^Hg. SPECT images were resampled to 199 × 199 × 397 arrays with the isotropic voxel size of 250.75 × 250.75 × 250.75 µm^3^, centered at the voxel (30, 30, 30) with the maximum value (i.e. the infusion/tumour site) and trimmed as 59 × 59 × 59 array with voxel value in Bq using MATLAB (R2024a, MathWorks, Natick, MA, USA). The distribution of ^197^Hg at 0 h p.i. was assumed to be the same as at 1 h p.i. The time integrated activity (Ã_0-7d_ Bq.s) from 0 h to 7 d p.i. of each voxel was calculated using the Trapezoid Rule for each pair of measured times (eg. 0-1h, 1h-1d, …4d-7d). The time integrated activity from 7 d p.i. to infinity (Ã_7d-∞_) of each voxel was calculated by dividing the activity (Bq) at 7 d p.i. by the decay constant of ^197^Hg (2.97 × 10^–6^ s^−1^). Ã_0d-∞_ is the sum of Ã_0-7d_ and Ã_7d-∞_. The voxel S-value array of the same configuration for ^197^Hg was generated by MCPN6 Ver. 6.1 (Los Alamos National Laboratory, Los Alamos, NM, USA) and MATLAB. ^197^Hg was assumed to be distributed homogeneously in the voxel (30, 30, 30). The detailed spectra of AE, internal conversion (IC) electrons, X-ray and γ-photons were taken from the MIRD Radionuclide Data (Eckerman and Endo, 2008) and included in the MCNP input file. The energy cutoffs for photons and electrons were both set as 20 eV. The energy deposition tally for both photons and electrons (*F8:p,e) was used to record the dose in MeV per starting particle to each voxel. This value was converted to Gy.Bq^−1^s^−1^ by multiplying by the unit conversion factor 1.602 × 10^–13^ J/MeV and the yield per decay of the respective particles (23.18, 0.8086, 26.38 and 0.1937 for AE, IC electrons, X- and γ-photons, respectively) which was divided by the mass of the voxel (1.6397 × 10^–8^ kg taking the brain grey/white matter density as 1.04 g/cm^3^) (ICRU-44). (Anonymous 1989). At least 1 × 10^7^ particles were launched for each simulation. The voxel dose array was obtained by convolving the Ã_0d-∞_ array with the S-value array. The absorbed dose maps surrounding the tumour in the brain, the maximum absorbed dose at the infusion site, the mean absorbed dose of the tumour (7 × 7 × 7 array) and in 0–1 mm (15 × 15 × 15–7 × 7 × 7), 1–3 mm (31 × 31 × 31–7 × 7 × 7) normal brain regions around the tumour edge, and histogram bin counts were generated from the dose array for a normalized CED of 1.0 MBq panitumumab-[^197^Hg]Hg-AuNPs or non-targeted [^197^Hg]Hg-AuNPs. The cumulative dose-volume-histogram (DVH) was calculated from the histogram bin counts.

### Statistical analysis

Data were expressed at mean ± SD. Statistical analyses were performed using Prism Ver. 10.3.1 software (GraphPad) with significance (*P* < 0.05) tested by a 2-tailed unpaired t-test or 1-way ANOVA with Tukey Multiple Comparisons Test.

## Results

### Synthesis and characterization of [^197^Hg]Hg-AuNPs

The incorporation of ^197^Hg into AuNPs during their synthesis by the Turkevich method was almost quantitative (98 ± 1%; n = 4). The specific activity of ^197^Hg-AuNPs was 1.4 × 10^–6^ Bq/AuNP. Reaction of [^197^Hg]Hg-AuNPs with a tenfold excess of panitumumab resulted in 0.4 ± 0.05 panitumumab conjugated per AuNP (n = 4) (see SI). Analysis of the shape and size of [^197^Hg]Hg-AuNPs by TEM revealed a mostly spherical or slightly oval shape with an average diameter of 26.8 ± 6.4 nm (n = 288) (Additional file 1: Fig. S4a,b). For comparison, TEM showed that AuNPs synthesized without incorporation of ^197^Hg were almost all spherical in shape with an average size of 18.2 ± 2.7 nm (n = 288) (Additional file 1: Fig. S4c,d). The size of panitumumab-[^197^Hg]Hg-AuNPs by TEM was not determined, but conjugation of one panitumumab IgG_2a_ to opposing aspects of a spherical AuNP would increase the diameter by a maximum of 29 nm, since the dimensions of one IgG molecule are 14.5 × 8.5 × 4.0 nm (Bagci et al. 1993). The UV–visible spectrum of [^197^Hg]Hg-AuNPs and AuNPs exhibited similar λ_max_ values (Additional file 1: Fig. S4e,f). There was no broadening of the absorption peak or shifting of the λ_max_ to a longer wavelength which may indicate AuNP aggregation. [^197^Hg]Hg-AuNPs demonstrated high stability at 37 °C for up to 144 h in artificial CSF or PBS, retaining > 97% of ^197^Hg but moderately lost up to 35% of ^197^Hg over this time period (~ 6% per d) when incubated in DMEM + 10% FBS (Additional file 1: Fig. S5). Loss of ^197^Hg from [^197^Hg]Hg-AuNPs in DMEM + 10% FBS may be due to the high concentration of glutathione in FBS (Bump & Reed, 1977) which could displace and bind ^197^Hg on the surface of the AuNPs.

**Fig. 1:**
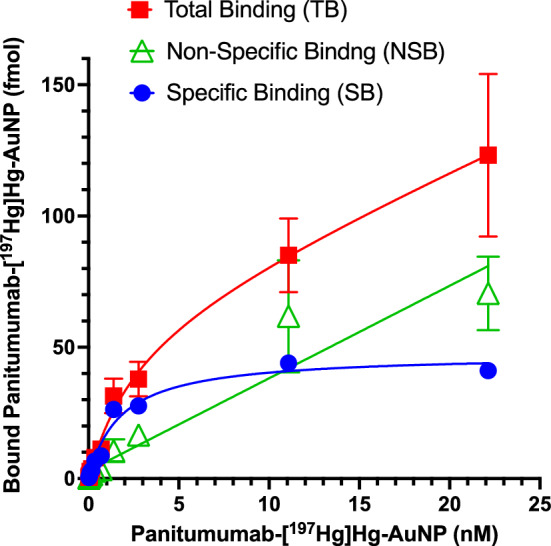
Binding of increasing concentrations of panitumumab-[^197^Hg]Hg-AuNPs to U251-Luc cells in the absence (total binding; TB) or presence of an excess of panitumumab (non-specific binding; NSB). Specific binding (SB) was calculated by subtracting NSB from TB. Fitting the SB curve to a one-site receptor binding model, the estimated K_D_ = 1.8 × 10^–9^ mol/L and B_max_ = 47.44 fmole, corresponding to 1.1 × 10^5^ EGFR/cell

The binding of increasing concentrations of panitumumab-[^197^Hg]Hg-AuNPs to U251-Luc cells in the absence or presence of 1,000-fold excess panitumumab is shown in Fig. 1. The TB of panitumumab-[^197^Hg]Hg-AuNPs to U251-Luc cells was decreased by competition with excess panitumumab (i.e. NSB) resulting in a SB curve that demonstrated saturable binding to EGFR. Based on the SB curve, the K_D_ = 1.8 × 10^–9^ mol/L and B_max_ = 47.44 fmole, corresponding to 1.1 × 10^5^ EGFR binding sites/cell, which was comparable to 2.3 × 10^5^ EGFR/cell estimated by flow cytometry (Additional file 1: Fig. S1).

### Cell binding and internalization studies

**Fig. 2:**
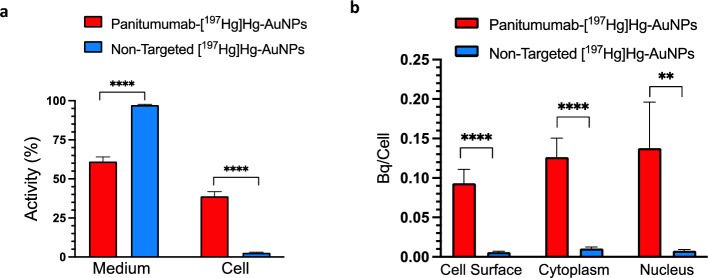
**a** Percent of activity remaining in the medium or cell-bound and **b** cell-bound activity (Bq/cell) present on the cell surface, in the cytoplasm or in the nucleus of U251-Luc human GBM cells incubated with panitumumab-[^197^Hg]Hg-AuNPs or non-targeted [^197^Hg]Hg-AuNPs for 19 h at 37 °C/5% CO_2_. Values are shown are the mean ± SD (n = 4) and significant differences are indicated by the asterisks: **(*P* = 0.0043), ****(*P* < 0.0001)

After incubation of U251-Luc cells with EGFR-targeted panitumumab-[^197^Hg]Hg-AuNPs (1 × 10^11^ AuNPs; 0.26 MBq) for 19 h at 37 °C the total cellular activity was 38.9 ± 2.9%, while non cell-bound activity remaining in the medium was 61.1 ± 2.9% (Fig. 2a). There was greater non cell-bound activity in the medium for U251-Luc cells incubated with non-targeted [^197^Hg]Hg-AuNPs (97.3 ± 0.4%; *P* < 0.0001) with only 2.7 ± 0.4% bound to U251-Luc cells, which was 14-fold lower than panitumumab-[^197^Hg]Hg-AuNPs (*P* < 0.0001). Panitumumab-[^197^Hg]Hg-AuNPs bound to the cell surface, internalized into the cytoplasm or in the nucleus were 0.093 ± 0.02 Bq/cell, 0.13 ± 0.02 Bq/cell and 0.14 ± 0.06 Bq/cell, respectively (Fig. 2b). In contrast, for non-targeted [^197^Hg]Hg-AuNPs, the activity on the cell surface, in the cytoplasm or in the nucleus (0.0059 ± 0.001 Bq/cell, 0.010 ± 0.002 Bq/cell and 0.0075 ± 0.002 Bq/cell, respectively) were significantly lower than panitumumab-[^197^Hg]Hg-AuNPs (*P* < 0.0001, *P* < 0.0001 and *P* = 0.0043, respectively). Further, studies of [^111^In]In-DOTA-panitumumab-AuNPs (Additional file 1: Fig. S6) showed selective binding to the cell surface and internalization into the cytoplasm and nucleus for EGFR-overexpressing U251-Luc cells compared to U87MG cells with low EGFR (Additional file 1: Fig. S1) or or murine GL261 glioblastoma cells with negligible EGFR (Guo et al. 2019).

### Clonogenic survival and γ-H2AX assays and cellular dosimetry

**Fig. 3:**
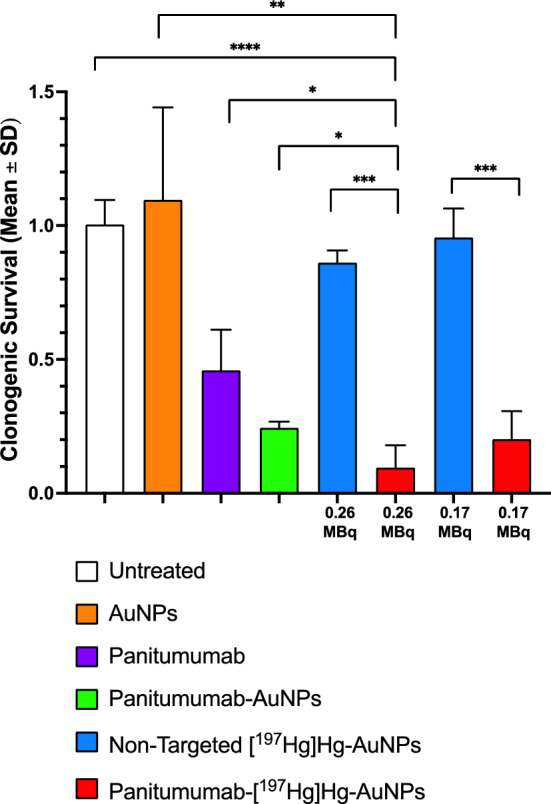
Clonogenic survival of U251-Luc cells treated in vitro for 24 h at 37 °C/5% CO_2_ with 0.17 or 0.26 MBq of panitumumab-[^197^Hg]Hg-AuNPs or non-targeted [^197^Hg]Hg-AuNPs or an equivalent amount of panitumumab, panitumumab-AuNPs, non-targeted AuNPs or no treatment, then seeded and cultured for 8 d. Values shown are the mean ± SD (n = 3) and significant differences are shown by the asterisks: *(*P* < 0.05), **(*P* < 0.01), *** (*P* < 0.001), ****(*P* < 0.0001)

Exposure of U251-Luc cells to 0.17 or 0.26 MBq (1.3 MBq/mL) of EGFR-targeted panitumumab-[^197^Hg]Hg-AuNPs (equivalent panitumumab concentration = 0.33 nM) for 24 h followed by culturing for 8 d decreased their survival fraction (SF) to 0.2 ± 0.1 and 0.1 ± 0.08, respectively (*P* = 0.240) compared to untreated cells (SF = 1.0 ± 0.09; Fig. 3). In contrast, the SF of U251-Luc cells treated with 0.17 or 0.26 MBq of non-targeted [^197^Hg]Hg-AuNPs were 0.96 ± 0.11 and 0.86 ± 0.05, respectively (*P* = 0.225), which was ninefold higher than panitumumab-[^197^Hg]Hg-AuNPs (*P* = 0.0002). The SF of U251-Luc cells exposed to non-radioactive AuNPs (1.10 ± 0.34) was not significantly different than untreated cells. Treatment with non-radioactive panitumumab-AuNPs or an equivalent amount of panitumumab decreased the SF to 0.24 ± 0.02 (*P* < 0.0001) and 0.46 ± 0.15 (*P* < 0.0001), respectively. However, the SF of U251-Luc cells treated with 0.26 MBq of panitumumab-[^197^Hg]Hg-AuNPs was twofold (*P* = 0.0408) and fivefold (*P* = 0.0142) significantly lower than exposure to non-radioactive panitumumab-AuNPs or an equivalent amount of panitumumab, respectively. Based on the subcellular distribution of panitumumab-[^197^Hg]Hg-AuNPs or non-targeted [^197^Hg]Hg-AuNPs in a U251-Luc cell with radius of cell (R_C_) = 11.8 ± 0.5 µm and radius of nucleus (R_N_) = 8.3 ± 0.2 µm (Additional file 1: Fig. S7) and exposure to activity in the medium (Additional file 1: Table S1), the absorbed doses in the nucleus in the clonogenic survival assays were 8.8 ± 2.9 Gy and 0.57 ± 0.2 Gy, respectively (Additional file 1: Table S2).

**Fig. 4:**
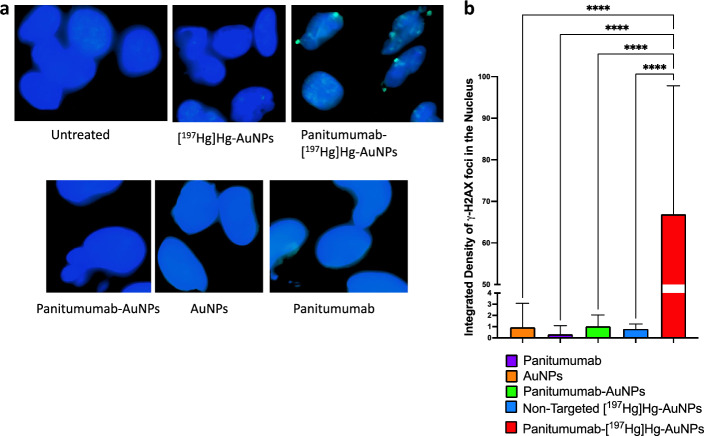
U251-Luc cells probed for DNA DSBs in the nucleus by immunofluorescence for γ-H2AX. **a** Images of the cell nucleus for untreated cells or cells treated for 24 h at 37 °C/5% CO2 with non-targeted [^197^Hg]Hg-AuNPs or panitumumab-[^197^Hg]Hg-AuNPs or an equivalent amount of non-radioactive panitumumab-AuNPs or AuNPs or panitumumab. γ-H2AX foci are noted as bright green foci in the nucleus which is counterstained blue with DAPI. **b** Quantification of the mean integrated density of γ-H2AX foci per nucleus area for each treatment group. Values are shown are the mean ± SD (n = 5) and significant differences are indicated by the asterisks: **** (*P* < 0.0001)

Immunofluorescence for γ-H2AX to probe DNA DSBs in the nucleus of U251-Luc cells revealed multiple foci for cells exposed to EGFR-targeted panitumumab-[^197^Hg]Hg-AuNPs but these were rare for treatment with non-targeted [^197^Hg]Hg-AuNPs (Fig. 4a). Untreated cells or cells exposed to non-radioactive panitumumab-conjugated or unconjugated AuNPs or an equivalent amount of panitumumab showed few γ-H2AX foci in the nucleus. Further analysis to quantify the density of γ-H2AX foci per nucleus area in a larger number of cells revealed that there was 84-fold significantly greater density of DNA DSBs in U251-Luc cells treated with panitumumab-[^197^Hg]Hg-AuNPs than in cells exposed to non-targeted [^197^Hg]Hg-AuNPs (67 ± 31 vs. 0.8 + 0.4; *P* = < 0.0001) (Fig. 4b). Cells treated with non-radioactive panitumumab-conjugated or unconjugated AuNPs or an equivalent amount of panitumumab exhibited very low γ-H2AX foci densities (1.1 ± 1.0, 0.96 ± 2.1, and 0.32 ± 0.77, respectively), which were all significantly lower than in cells treated with panitumumab-[^197^Hg]Hg-AuNPs (*P* < 0.0001).

### SPECT/CT imaging and biodistribution studies

**Fig. 5:**
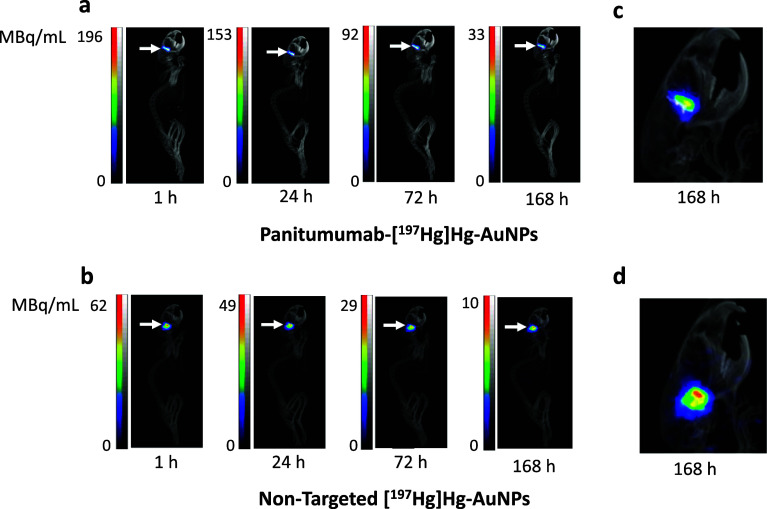
SPECT/CT images of NRG mice with orthotopic U251-Luc human GBM tumours in the right hemisphere of the brain administered **a** panitumumab-[^197^Hg]Hg-AuNPs or **b** non-targeted [^197^Hg]Hg-AuNPs by CED (arrows) at selected times up to 168 h post-infusion (p.i.). Intensity scales (MBq/mL) are shown for each timepoint post-infusion. Magnified view of the head showing differences in local diffusion from the infusion site in the brain for **c** panitumumab-[^197^Hg]Hg-AuNPs or **d** non-targeted [^197^Hg]Hg-AuNPs

**Fig. 6:**
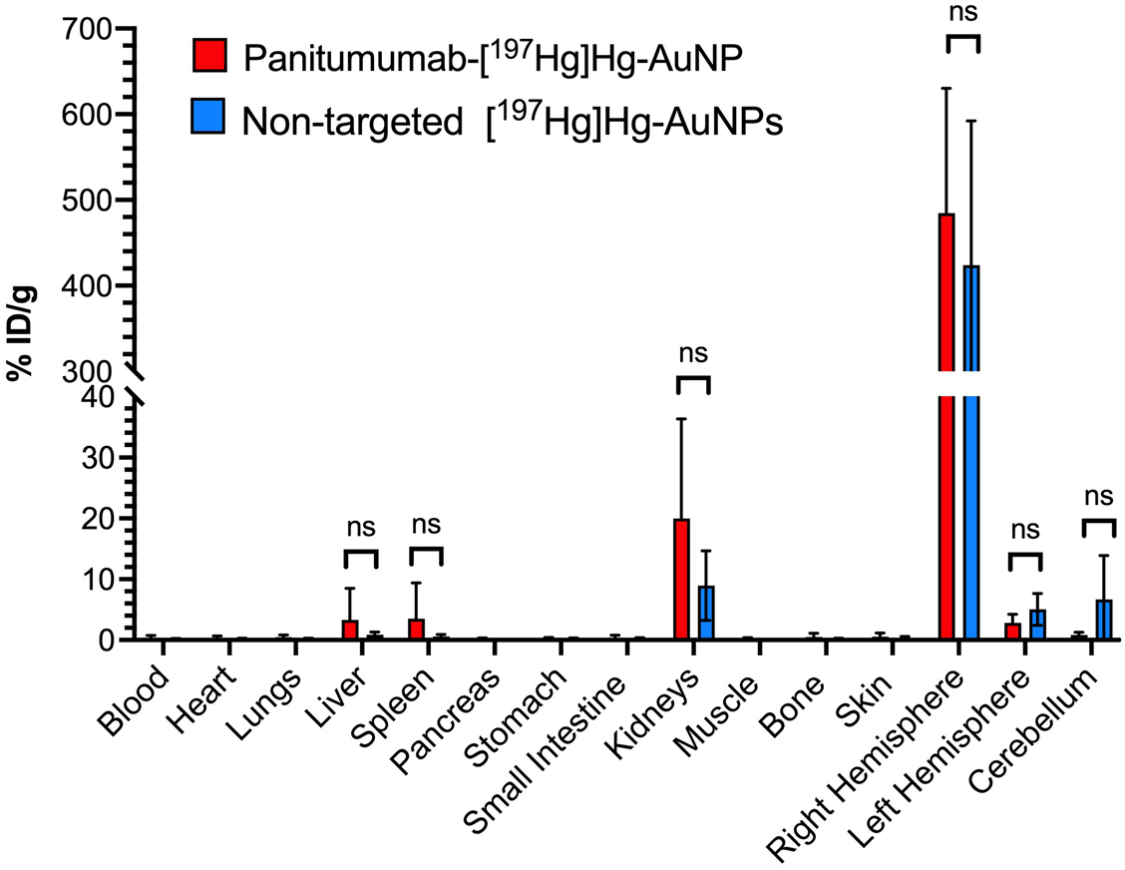
Biodistribution of panitumumab-[^197^Hg]Hg-AuNPs or non-targeted [^197^Hg]Hg-AuNPs at 168 h p.i. The tumour could not be excised from the brain and was included in the right hemisphere tissue while the left hemisphere and cerebellum were non-tumour bearing. Values shown are the mean ± SD (n = 4–5). There were no significant differences (*P* > 0.05) in the biodistribution of panitumumab-[^197^Hg]Hg-AuNPs and non-targeted [^197^Hg]Hg-AuNPs at this timepoint

SPECT/CT images post-CED infusion of EGFR-targeted panitumumab-[^197^Hg]Hg-AuNPs or non-targeted [^197^Hg]Hg-AuNPs demonstrated intense focal retention at the site of infusion in the brain in NRG mice with U251-Luc tumors in the right cerebral hemisphere at all time points up to 7 d (Fig. 5a,b). There was no activity observed in other regions of the brain or in other normal organs. However, closer examination of the images revealed apparently greater diffusion of non-targeted [^197^Hg]Hg-AuNPs from the site of CED than panitumumab-[^197^Hg]Hg-AuNPs in these mice (Fig. 5c,d) as well as two additional mice in each group (Additional file 1: Fig. S8). This was further noted in the excised brain by a darker colour imparted by the AuNPs (Additional file 1: Fig. S9). Biodistribution studies (n = 4–5) at 7 d post-infusion determined that there was 484.5 ± 145.6% ID/g and 423.9 ± 168.3% ID/g, respectively in the tumour-bearing right cerebral hemisphere for panitumumab-[^197^Hg]Hg-AuNPs and non-targeted [^197^Hg]Hg-AuNPs (*P* = 0.5814; Fig. 6). Activity in the tumour-bearing right hemisphere for panitumumab-[^197^Hg]Hg-AuNPs was 172-fold and 579-fold significantly higher than in the normal left hemisphere (2.8 ± 1.4% ID/g; *P* < 0.0001) and cerebellum (0.8 ± 0.4% ID/g; *P* < 0.0001). Similarly, activity in the tumour-bearing right hemisphere for non-targeted [^197^Hg]Hg-AuNPs was 85-fold and 63-fold significantly higher than in the normal left hemisphere (5.0 ± 2.6% ID/g; *P* < 0.0001) and cerebellum (6.7 ± 7.2% ID/g; *P* < 0.0001). There were no significant differences (*P* > 0.05) in activity in the left cerebral hemisphere or cerebellum between panitumumab-[^197^Hg]Hg-AuNPs and non-targeted [^197^Hg]Hg-AuNPs. The whole right brain retained 71.1 ± 17.5% and 64.7 ± 23.8% of panitumumab-[^197^Hg]Hg-AuNPs and non-targeted [^197^Hg]Hg-AuNPs, respectively (*P* = 0.644). Uptake of panitumumab-[^197^Hg]Hg-AuNPs in the liver, spleen and kidneys were 3.3 ± 5.2% ID/g, 3.5 ± 5.9% ID/g and 20.0 ± 16.3% ID/g, which were 147-fold, 138-fold and 24-fold significantly lower than in the tumour-bearing right hemisphere. Similarly, the uptake of non-targeted [^197^Hg]Hg-AuNPs in the liver, spleen and kidneys were 0.9 ± 0.4, 0.6 ± 0.3, 8.9 ± 5.7% ID/g, respectively, which were 471-fold, 707-fold and 48-fold significantly lower than in the tumour-bearing right hemisphere. All other normal organs accumulated < 0.5% ID/g of panitumumab-[^197^Hg]Hg-AuNPs or non-targeted [^197^Hg]Hg-AuNPs.

### Estimation of radiation absorbed doses and dose mapping

**Fig. 7:**
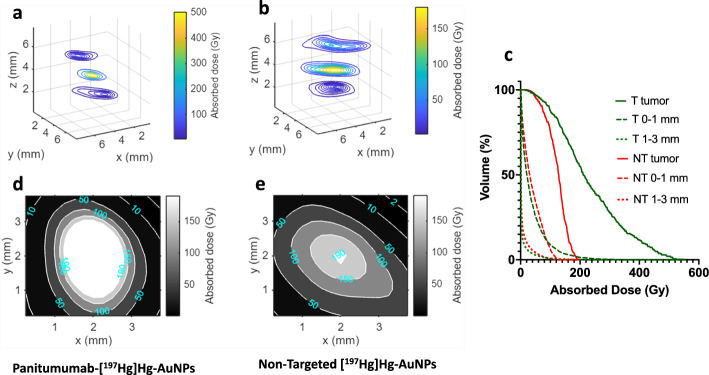
Absorbed dose distribution around the intratumoural infusion site in the brain for NRG mice administered panitumumab-[^197^Hg]Hg-AuNPs (**a**, **d**) or non-targeted [^197^Hg]Hg-AuNPs (**b**, **e**) by CED. Panels **a** and **b** show the 3D isodose contour x–y slices through the infusion site at z = 4 mm and at 1 mm away from the tumour edge at z = 2 or z = 6 mm. Panels **d**, **e** show the 2D isodose contour for an x–y cross-section through the z-slice of the tumour that exhibited the maximum absorbed dose. Panel **c** shows the DVH for the tumour and surrounding brain regions at 0–1 mm and 1–3 mm from the tumour edge for either panitumumab-[^197^Hg]Hg-AuNPs (T) or non-targeted [^197^Hg]Hg-AuNPs (NT)

The absorbed dose distribution around the infusion site/tumour after CED of 1.0 MBq of panitumumab-[^197^Hg]Hg-AuNPs or non-targeted [^197^Hg]Hg-AuNPs was mapped based on the serial SPECT/CT images and MCNP simulated S-value array for ^197^Hg and is shown as isodose contour x–y slices through the infusion site at z = 4 mm and 1 mm away from the tumour edge at z = 2 and z = 6 mm (Fig. 7a,b), and as enlarged x–y cross sections through the z-slice with the maximum absorbed dose (Fig. 7d,e). The cumulative DVH for the tumour, and healthy tissue regions within 0–1 mm or 1–3 mm of the tumour edge is shown in Fig. 7c. The DVH showed that 58% of the tumour received > 190 Gy after CED of 1.0 MBq panitumumab-[^197^Hg]Hg-AuNPs but only 0.6% of the tumour treated with 1.0 MBq of non-targeted [^197^Hg]Hg-AuNPs received this dose. Nonetheless, both 1.0 MBq of panitumumab-[^197^Hg]Hg-AuNPs and non-targeted [^197^Hg]Hg-AuNPs delivered > 50 Gy to 97% and 95% of the tumour, respectively, and 50% of the tumour received > 210 Gy and > 128 Gy, respectively. In contrast, the absorbed dose in the healthy brain margin surrounding the tumour decreased dramatically as the distance increased. CED of 1.0 MBq of panitumumab-[^197^Hg]Hg-AuNPs delivered 550, 234, 37 and 1.7 Gy to the infusion point, tumour, 0–1 mm region and 1–3 mm region from the tumour edge, respectively. CED of 1.0 MBq of non-targeted [^197^Hg]Hg-AuNPs delivered 197, 122, 39 and 3.3 Gy to the infusion point, tumour, 0–1 mm region and 1–3 mm region from the tumour edge, respectively.

## Discussion

We report here the synthesis of novel AE-emitting radiation nanomedicines for treatment of GBM composed of panitumumab-conjugated [^197^Hg]Hg-AuNPs or non-targeted [^197^Hg]Hg-AuNPs. The cytotoxicity and DNA-damaging properties of these agents and their cellular dosimetry were studied in vitro using U251-Luc human GBM cells and their tumour and normal tissue localization and dosimetry in vivo were evaluated after CED in NRG mice with U251-Luc human GBM tumours in the brain. [^197^Hg]Hg-AuNPs were produced by integrating ^197^Hg into AuNPs synthesized by the Turkevich method (Kimling et al., 2006). These [^197^Hg]Hg-AuNPs were then conjugated to panitumumab to target EGFR overexpressed on GBM cells (Heimberger et al. 2005) or remained non-targeted. Droop et al. (Droop et al. 2023) described an alternative method for synthesis of [^197^Hg]Hg-AuNPs involving tannic acid reduction of a dissolved gold target following irradiation via the ^197^Au(p,n)^197m^Hg/^197^Hg reaction, but these [^197^Hg]Hg-AuNPs were not targeted and no studies of their cytotoxicity in vitro or tumour and normal tissue biodistribution or dosimetry in vivo were performed. In our study, the morphology and size distribution of [^197^Hg]Hg-AuNPs assessed by TEM were slightly different than AuNPs (Additional file 1: Fig. S4). While AuNPs were spherical with a mean diameter of 18.2 ± 2.7 nm, [^197^Hg]Hg-AuNPs were larger (26.8 ± 6.4 nm) and more oval in shape. The larger size of [^197^Hg]Hg-AuNPs may be due to the lower pH under which they were synthesized, since ^197^Hg is separated from a gold target (Additional file 1: Fig. S2a) using a LN column eluted with 6 M HCl (Chen et al. 2023). Although multiple washes with Milli-Q® water were performed, trace amounts of 6M HCl remain, which results in pH = 4 in the synthesis reaction. The size, polydispersity and morphology of AuNPs produced by the Turkevich method is influenced by pH (Patungwasa and Hodak 2008). In particular, pH < 5 results in larger AuNPs with greater polydispersity (Tyagi et al. 2016). Synthesis at pH 5.0–5.5 yields AuNPs with oblate shape (Patungwasa and Hodak 2008). In our study, AuNPs without ^197^Hg were synthesized at pH of 6–7, which yielded spherical, smaller and more uniform AuNPs. Integration of ^197^Hg into AuNPs was almost quantitative (98 ± 1%) and [^197^Hg]Hg-AuNPs were stable in vitro to loss of ^197^Hg up to 7 d at 37 °C/5% CO_2_ in artificial human CSF (Additional file 1: Fig. S5). Lower stability was observed in DMEM + 10% FBS. FBS contains high concentrations of glutathione, cysteine and albumin that bind mercury (Song et al. 2021) and may displace ^197^Hg from the surface of [^197^Hg]Hg-AuNPs. Although lower stability was observed in DMEM + 10% FBS, CSF is most clinically relevant since [^197^Hg]Hg-AuNPs will be administered by CED into tumours in the brain, avoiding exposure to the plasma, which is more relevant for i.v. injection. Integration of radiometals into AuNPs offers advantages in terms of higher radiochemical yield, simplicity of synthesis and stability compared to complexation by chelators conjugated to AuNPs (Daems et al. 2021).

To target EGFR overexpressed on U251-Luc human GBM cells (Additional file 1: Fig. S1), [^197^Hg]Hg-AuNPs were conjugated to panitumumab through forming a gold-thiol bond with a cysteine in panitumumab as reported for conjugation of EGF to AuNPs (Song et al., 2017). Free cysteines are present in some recombinant monoclonal antibodies (Metcalfe 2022). Panitumumab-[^197^Hg]Hg-AuNPs exhibited high affinity binding to EGFR on U251-Luc cells (Fig. 1) with a K_D_ = 1.8 × 10^–9^ mol/L comparable to [^177^Lu]Lu-DOTA-panitumumab (1.0 × 10^–9^ mol/L) binding to EGFR-positive MDA-MB-468 human breast cancer cells (Aghevlian et al. 2018). Panitumumab conjugation increased binding of [^197^Hg]Hg-AuNPs to U251-Luc human GBM cells in vitro vs. non-targeted [^197^Hg]Hg-AuNPs and promoted internalization into the cytoplasm and importation into the nucleus (Fig. 2). Nuclear localization may be mediated by a nuclear localization sequence (NLS) in the EGFR (Lo et al. 2006). Internalization and nuclear uptake are important to maximize the DNA-damaging properties of AE (Ku et al. 2019). Panitumumab-[^197^Hg]Hg-AuNPs were ninefold more effective at reducing the SF of U251-Luc cells than non-targeted [^197^Hg]Hg-AuNPs (Fig. 3). This greater cytotoxic potency of panitumumab-[^197^Hg]Hg-AuNPs was correlated with an 84-fold greater density of DNA DSBs in the nucleus of U251-Luc cells vs. non-targeted [^197^Hg]Hg-AuNPs measured by immunofluorescence for γ-H2AX (Fig. 4). These results revealed the importance of panitumumab conjugation for increasing the binding, internalization and nuclear uptake of [^197^Hg]Hg-AuNPs by U251-Luc cells, which rendered them more cytotoxic than non-targeted [^197^Hg]Hg-AuNPs. Non-radioactive panitumumab-AuNPs or an equivalent amount of panitumumab were cytotoxic to U251-Luc cells but these were twofold and fivefold less potent, respectively, than panitumumab-[^197^Hg]Hg-AuNPs (Fig. 3) and did not cause DNA DSBs (Fig. 4). Unlabeled AuNPs were not cytotoxic and did not damage DNA. Although EGFR are overexpressed on 50–60% of GBM (Heimberger et al. 2005), panitumumab alone proved ineffective for treating GBM in a recently reported clinical trial (Spiekman et al. 2024). Nonetheless, panitumumab is a potent inhibitor of EGFR-mediated growth signaling (Saltz et al. 2006) and EGFR remains a potential therapeutic target in GBM (Kalman et al. 2013).

SPECT/CT imaging and biodistribution studies showed that panitumumab-[^197^Hg]Hg-AuNPs and non-targeted [^197^Hg]Hg-AuNPs were strongly retained at the site of infusion in the tumour-bearing right hemisphere of the brain in NRG mice with U251-Luc tumours (Fig. 5a,b), with very low redistribution to the healthy brain and low uptake in other normal organs (Fig. 6). However, images of non-targeted [^197^Hg]Hg-AuNPs suggested greater diffusion from the infusion site than panitumumab-[^197^Hg]Hg-AuNPs (Fig. 5c,d and Additional file 1: Fig. S8). We hypothesize that the lower diffusion of panitumumab-[^197^Hg]Hg-AuNPs from the site of infusion could be due to in vivo binding and internalization by U251-Luc cells, but this has not yet been determined. The strong retention of panitumumab-[^197^Hg]Hg-AuNPs and non-targeted [^197^Hg]Hg-AuNPs in the brain after CED agrees with our previous report of [^177^Lu]Lu-MCP-AuNPs administered by CED to NRG mice with U251-Luc tumours (Georgiou et al. 2023). AuNPs provide an “anchoring” effect that prevents their redistribution after CED. This has been shown by others for ^64^Cu-labeled AuNPs (Amirrashedi et al. 2024). The retention of panitumumab-[^197^Hg]Hg-AuNPs after CED of 1 MBq resulted in very high absorbed doses at the site of infusion, resulting in 58% of the tumour receiving > 190 Gy based on the DVH (Fig. 7c). Due to greater local diffusion, only 0.6% of the tumour received this dose for non-targeted [^197^Hg]Hg-AuNPs, but 95% of the tumour still received a dose of > 50 Gy. Patients with GBM are treated with 50–60 Gy of X-radiation in 30 × 2 Gy fractionated doses (Barani and Larson 2015). The incidence of radiation necrosis in the brain in humans over 1 − 2 years treated with XRT is 5% at a cumulative dose of 120 Gy and 10% at 150 Gy, when administered as fractionated doses of < 2.5 Gy (Lawrence et al. 2010). The dose deposited by panitumumab-[^197^Hg]Hg-AuNPs and non-targeted [^197^Hg]Hg-AuNPs after CED of 1 MBq was highly conformal, decreasing to 37 and 39 Gy in a 0–1 mm region from the tumour edge, but dramatically to 1.7 and 3.3 Gy in a 1–3 mm region from the tumour edge (Fig. 7a-e). Thus, we predict that therapeutic and safe doses could potentially be delivered to GBM tumours in patients by CED of panitumumab-[^197^Hg]Hg-AuNPs or non-targeted [^197^Hg]Hg-AuNPs.

## Conclusions

Radiation nanomedicines for treatment of GBM were synthesized by integrating the AE-emitter, ^197^Hg into AuNPs which were subsequently conjugated to panitumumab to target EGFR overexpressed on human GBM cells or remained non-targeted. Panitumumab-[^197^Hg]Hg-AuNPs exhibited greater binding, internalization and nuclear uptake in U251-Luc human GBM cells, which increased DNA DSBs and their cytotoxicity in vitro vs. non-targeted [^197^Hg]Hg-AuNPs. Both panitumumab-[^197^Hg]Hg-AuNPs and non-targeted [^197^Hg]Hg-AuNPs were retained at an intratumoural infusion site in the brain after CED in NRG mice, depositing high doses in U251-Luc tumours but dramatically lower doses at 1–3 mm from the tumour edge in healthy brain. These radiation nanomedicines, particularly panitumumab-[^197^Hg]Hg-AuNPs due to their higher cytotoxicity in vitro are promising for treatment of GBM via CED and this approach may ultimately improve patient outcome.

## Supplementary Information

**Additional file 1**. Supplementary Information (SI).

## Data Availability

All data generated or analyzed during this study are included in this published article [and its Additional file 1].

## Abbreviations

Ã: Time integrated activity
AE: Auger electron
AuNP: Gold nanoparticle
BBB: Blood Brain Barrier
BLI: Bioluminescence imaging
BSA: Bovine serum albumin
CED: Convection Enhanced Delivery
CO_2_: Carbon Dioxide
CSF: Cerebral Spinal Fluid
CT: Computed tomography
DAPI: 4′,6-Diamidino-2-phenylindole
DMEM: Dulbecco's Modified Eagle Medium
DLS: Dynamic Light Scattering
DNA: Deoxyribonucleic acid
DOTA: 1,4,7,10-Tetraazacyclododecane-1,4,7,10-tetraacetic acid
DSB: Double Strand Breaks
DVH: Dose volume histogram
EGFR: Epidermal Growth Factor Receptor
FBS: Fetal Bovine Serum
GBM: Glioblastoma Multiforme
HAuCl_4_: Tetrachloroauric (III) acid
HCl: Hydrochloric acid
HgCl_2_: Mercury (II) chloride
HNO_3_: Nitric acid
IC: Internal conversion
i.v.: Intravenous
LET: Linear Energy Transfer
LN: Lanthanide resin
MCNP: Monte Carlo N-particle
MIRD: Medical Internal Radiation Dose
NaI(TI): Sodium iodide with thallium dopant
NLS: Nuclear localization sequence
NRG: NOD-*Rag1*^*null*^* IL2rg*^*null*^
NSB: Non-specific binding
O_2_: Oxygen gas
PBS: Phosphate-buffered saline
PD1: Program cell death-1
PE: Plating efficiency
PEG_2k_-LA: Lipoic acid-polyethylene glycol 2kDa
p.i.: Post-infusion
RT: Room temperature
ROS: Reactive Oxygen Species
SB: Specific binding
SF: Survival fraction
SPECT: Single photo emission computed tomography
TB: Total binding
TEM: Transmission Electron Microscopy
TMZ: Temozolomide
Tween-20: Polyoxyethylene (20) sorbitan monolaurate
XRT: Radiation Therapy
%ID/g: Percent injected dose per gram
λ_max_: Absorption peak
γ-H2AX: Phosphorylated histone-2AX
