# Supplementary material for: Auger electron-emitting EGFR-targeted and non-targeted [197Hg]Hg-gold nanoparticles for treatment of glioblastoma multiforme (GBM)
Source: EJNMMI Radiopharm Chem. 2025 Jul 17;10:45. doi: 10.1186/s41181-025-00367-2 (PMC12271031; doi:10.1186/s41181-025-00367-2)
Supplement: Supplementary file 1 — Additional file 1. Supplementary Information (SI). [file 41181_2025_367_MOESM1_ESM.docx]

**Auger Electron-Emitting EGFR-Targeted and Non-Targeted [^197^Hg]Hg-Gold Nanoparticles for Treatment of Glioblastoma Multiforme (GBM)**

Madeline K. Brown ^1^, Zhongli Cai ^1^, Constantine J. Georgiou ^1^, Shaohuang Chen ^2,3^, Yumeela Ganga-Sah ^2^, Valery Radchenko ^2,4,^, James T. Rutka ^5,6^, Raymond M. Reilly ^1,7,8^ *

^1^ Department of Pharmaceutical Sciences, University of Toronto, Toronto, ON, Canada

^2^ Life Sciences Division, TRIUMF, Vancouver, BC, Canada

^3^ Department of Chemistry, Simon Fraser University, Vancouver, BC, Canada

^4^ Department of Chemistry, University of British Columbia, Vancouver, BC, Canada

^5^ Division of Neurosurgery, The Hospital for Sick Children, Toronto, ON, Canada

^6^ Division of Neurosurgery, Department of Surgery, Temerty Faculty of Medicine, University of Toronto, Toronto, ON, Canada

^7^ Department of Medical Imaging, University of Toronto, Toronto, ON, Canada

^8^ Princess Margaret Cancer Centre, University Health Network, Toronto, ON, Canada

**Supplementary Information**

* Correspondence to: Raymond M Reilly; Email: [raymond.reilly@utoronto.ca](about:blank)

Leslie Dan Faculty of Pharmacy, University of Toronto, Toronto, ON, Canada M5S 3M2

Tel. 1-416-946-5522; FAX: 1-416-978-8511

**Estimation of the EGFR Expression of U251-Luc Cells**

The EGFR expression of U251-Luc cells was estimated based on the relative mean fluorescence intensity by flow cytometry analysis compared to MDA-MB-468 and MDA-MB-231 human breast cancer cells which express 2.4 × 10^6^ and 5.4 × 10^5^ EGFR/cell respectively (Reilly et al., 2000). Cells were harvested from 175T flask containing DMEM + 10% FBS. Cells were rinsed once with 5 mL of fluorescence-activated cell sorting (FACS) buffer (0.1% NaN_3_ and 1% BSA in PBS), then pelleted by centrifugation at 150 × g for 5 min. Cells were resuspended in FACS buffer and 4 × 10^5^ live cells were dispensed into 1.5 mL Eppendorf tubes, then pelleted by centrifugation at 400 × g for 5 min. Pelleted cells in 100 μL of FACS buffer were stained with panitumumab or Alexa Fluor 488-hIgG in FACS buffer (100 μL) for 30 minutes on ice. The tubes were protected from light by wrapping in aluminum foil while gently shaking on a bench-top incubator-shaker at 150 rpm. Cells were then centrifuged at 400 × *g* for 5 min at 4 °C and cell pellets were resuspended in 200 μL of ice cold FACS buffer. The resuspended cells were filtered into tubes with a cell strainer cap 12x75mm (5mL, round bottom, polystyrene, sterile, Falcon 352235) and were analyzed using a CytoFLEX S flow cytometer (Beckman Coulter). Based on this analysis (**Fig. S1**) the EGFR expression of U251-Luc cells was estimated as 2.3 × 10^5^ receptors/cell and was higher than U87MG human GBM cells (8 × 10^4^ EGFR/cell).

**Fig. S1.** Flow cytometric analysis for EGFR expression for MDA-MB-468, MDA-MB-231, U251-Luc and U87MG cells.

**Production and Decay Scheme of ^197m^Hg/^197^Hg**

^197^Hg (t_1/2_=64.1 h) and ^197m^Hg (t_1/2_=23.8 h) were co-produced by the ^197^Au(p,n)^197m^Hg/^197^Hg reaction by irradiation of an isotopically pure (100%) natural gold (^197^Au) target (**Fig. S2a**) in a TR13 (13 MeV) cyclotron on a tantalum backing at TRIUMF (University of British Columbia, Vancouver, BC, Canada) (Randhawa et al., 2021). The target was irradiated with 12.8 MeV protons at a beam current of 30 μA for up to 4 h. The target was then dissolved in aqua regia [2.20 mL of concentrated (12.2 M) HCl (Sigma-Aldrich, St. Louis, MO, USA) and 0.426 mL of concentrated (15.7 M) HNO_3_ (Sigma-Aldrich, St ) at 120 ^o^C for 1 h, followed by filtration, then two rinses with 1 mL of 6 M HCl. ^197^Hg/^197m^Hg were separated from ^197^Au using an LN resin (Chen et al., 2023). ^197^Hg/^197m^HgCl_2_ was eluted in 5 mL of 6 M HCl, then concentrated under vacuum, rinsed twice with 1 mL of Milli-Q® water, with each wash reconcentrated, before recovery as [^197^Hg]/[^197m^Hg]HgCl_2_ in 170 μL of Milli-Q® water. [^197^Hg]/[^197m^Hg]HgCl_2_ was shipped overnight from TRIUMF to the University of Toronto. The activity and concentration on receipt were 114 MBq and 1027 MBq/mL. ^197m^Hg (t_1/2_=23.8 h) decays to ^197^Hg then to stable ^197^Au, emitting 19 AEs/decay (**Fig. S2b**), while ^197^Hg (_t1/2_=64.1 h) decays to ^197^Au, emitting 23 AEs/decay (Ku et al., 2019).

**Fig. S2. a.** Gold target used to produce ^197m^Hg/^197g^Hg by the ^197^Au(p,n)^197m^Hg/^197g^Hg reaction. **b.** Decay scheme of ^197m^Hg and ^197g^Hg.

**Characterization of [^197^Hg]Hg-AuNPs**

[^197^Hg]Hg-AuNPs were characterized for size and shape by transmission electron microscopy (TEM) and aggregation was assessed by UV-visible spectroscopy. A 50 μL sample of decayed [^197^Hg]Hg-AuNPs was mounted on copper grids and TEM images were acquired using a Hitachi HT7700 Transmission Electron Microscope equipped with an XR280 AMT camera system at 100 kV and 30,000 × magnification. The TEM images were processed and analyzed using ImageJ (Version 1.53e, National Institutes of Health, Bethesda, MD, USA) with a macro to estimate particle diameters. The macro included steps of setting auto threshold to define particle areas, then analyzing the area of the particles. The particles were assumed to be spheres, and the diameters were calculated using the formula of 2 × (area/π)^1/2^. The distribution of particle sizes was plotted and the mean ± SD particle size calculated. A UV-visible spectrum of decayed [^197^Hg]Hg-AuNPs was obtained on an Ultraspec 3100 Pro spectrophotometer (GE Healthcare/Amersham Pharmacia, UK) from 200 nm to 700 nm to identify the characteristic absorbance peak (λ_max_) of AuNPs of this size (24 nm AuNP λ_max_= 524 nm and 33 nm AuNPs λ_max_= 528 nm) (He et al., 2005) and to detect aggregated AuNPs which show a broadened and shifted peak at 600-700 nm. The stability of [^197^Hg]Hg-AuNPs was evaluated in Dulbecco’s Modified Eagles Medium (DMEM) containing 10% fetal bovine serum (FBS; Gibco-Invitrogen, Massachusetts, USA), phosphate-buffered saline pH 7.4 (PBS; Gibco, ThermoFisher Scientific, Paisley, UK) and artificial cerebral spinal fluid (CSF; Product BZ178, Biochemazone, Leduc, Alberta, CA). For these assays, 1 mL of [^197^Hg]Hg-AuNPs (1 × 10^11^ AuNPs) were mixed with 1 mL of medium + 10% FBS, PBS or artificial CSF and incubated at 37 ^o^C/5% CO_2_. Samples were obtained at 1, 3, 5, 24, 48, 72 and 144 h and free ^197^Hg separated from [^197^Hg]Hg-AuNPs by ultracentrifugation at 15,000 × g for 15 mins and measured in a radioisotope dose calibrator.

**Labeling of panitumumab with ^111^In**

Panitumumab was conjugated to DOTA and labeled with ^111^In as previously reported (Facca et al., 2022). Briefly, panitumumab (Vectibix®, Amgen, Mississauga, ON, Canada) was buffer-exchanged into ddH_2_0 and concentrated to 1-2 mg/mL by ultrafiltration on a Amicon ultra 0.5 mL device (MW cut-off = 30 kDa; Amicon). Panitumumab (200 μg; 10 μL) was then reacted with a 20-fold molar excess of tetraazacyclododecane-1,4,7,10-tetraacetic acid N-hydroxysuccinimide ester N-hydroxysuccinimide ester (DOTA-NHS; Macrocyclics, Plano, TX), purified and buffer-exchanged and then concentrated to 17.3 mg/mL into ddH_2_0, by ultrafiltration on a 30 kDa Amicon ultra 0.5 mL device (Amicon, Sigma-Aldrich, St. Louis, MO). DOTA-panitumumab (33.3 μg) was labeled with ^111^In by incubation with [^111^In]InCl_3_ (BWXT, Vancouver, BC, Canada) in 1M NaAc buffer, pH 6.1 at 42.5 ^o^C for 3 h. The final RCP was 95% measured by instant thin layer-silica gel chromatography (ITLC-SG) developed in 0.1M Na citrate buffer, pH5.0 (R_f_ of [^111^In]In-DOTA-panitumumab = 0.0; R_f_ of [^111^In]In-DOTA or free ^111^In = 1.0).

**Evaluation of panitumumab conjugation to AuNPs**

To study the effect of increasing panitumumab conjugation to AuNPs on their binding and internalization into EGFR-positive U251-Luc human GBM cells, 1 × 10^11^ AuNPs in 1 mL ddH_2_0 were incubated with a 10-250 fold excess of panitumumab (1-25 μg) that incorporated [^111^In]In-DOTA-panitumumab (0.0072 MBq/μg) to radiotrace and quantify the number of panitumumab bound to AuNPs. The conjugation conditions are described in the main manuscript and labeling of panitumumab with ^111^In is described above. Once the panitumumab-AuNPs were isolated by ultracentrifugation at 10,000 × g for 15 mins, the fraction of [^111^In]In-DOTA-panitumumab bound was measured in a radioisotope dose calibrator (CRC-15R, Capintec Inc, Ramsey, NJ). The number of panitumumab conjugated to AuNPs was calculated by multiplying the fraction of [^111^In]In-DOTA-panitumumab bound based on the dose calibrator measurements by the excess of panitumumab reacted with the AuNPs. To further evaluate panitumumab-AuNPs, U251-Luc cells were incubated for 19 h at 37 ^o^C/5% CO_2_ with [^111^In]In-DOTA-panitumumab-AuNPs prepared at different reaction ratios of panitumumab:AuNP and the percentage of cell-bound and internalized activity was measured as described in the main manuscript. The reaction ratio (fold excess) that provided the highest cell-binding and internalization was selected as the optimum condition for panitumumab conjugation of AuNPs.

The number of panitumumab conjugated per AuNP was 0.4 ± 0.05, 2.0 ± 0.3, 3.1 ± 0.4, 3.8 ± 0.3, 4.9 ± 0.6, and 6.4 ± 0.6 when AuNPs were reacted with panitumumab at a 10, 50, 100, 150 or 200-fold excess. When [^111^In]In-DOTA-panitumumab-AuNPs were incubated with U251-Luc cells, the percentage of the activity incubated with the cells that was cell-bound and internalized decreased as the reaction ratio (fold excess) of panitumumab was increased (**Fig. S3a**). However, there was no significant decrease in the percentage of the cell-bound activity that was internalized into the cytoplasm and nucleus as the reaction ratio was increased (**Fig. S3b**). Based on these results, a reaction ratio of 10-fold excess of panitumumab was selected to conjugate panitumumab to [^197^Hg]Hg-AuNPs.

**Fig. S3. a.** Percentage of total activity of [^111^In]In-DOTA-panitumumab-AuNPs incubated with U251-Luc cells that was internalized into the cytoplasm + nucleus or bound to the cell surface vs. reaction ratio of panitumumab/AuNPs. **b.** Percentage of the total cell-bound activity that was internalized into the cytoplasm + nucleus or bound to the cell surface vs. reaction ratio of panitumumab/AuNPs.

**Fig. S4.** TEM images and size distribution of decayed [^197^Hg]Hg-AuNPs (**a,b**) or non-radioactive AuNPs (**c,d**). UV-visible spectra of [^197^Hg]Hg-AuNPs (**e**) or non-radioactive AuNPs (**f**). Dotted line indicates the λ_max_ values.

**
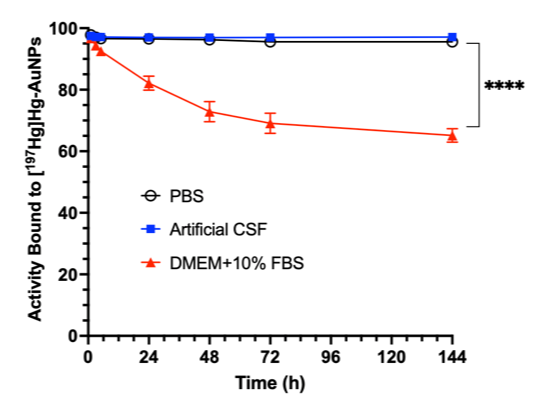
**

**Fig. S5.** Stability to loss of ^197^Hg of [^197^Hg]Hg-AuNPs *in vitro* in PBS, artificial CSF or DMEM+10% FBS incubated up to 144 h at 37 ^o^C/5% CO_2_. Values shown are the mean ± SD (n=4). Significant differences at 144 h between PBS or artificial CSF vs. DMEM+10% FBS are shown by the asterisks: ****(*P*<0.0001).

**Binding and internalization of panitumumab-[^197^Hg]Hg-AuNPs in GBM cells with different EGFR expression levels**

Using a 10:1 reaction ratio of panitumumab:AuNPs resulting in 0.4 ± 0.05 panitumumab conjugated per AuNP, [^111^In]In-DOTA-panitumumab-AuNPs were constructed and used to measure the binding and internalization in U251-Luc human GBM cells with high EGFR expression or U87MG cells with low EGFR (**Fig. S1**) or murine GL261 glioblastoma cells with negligible EGFR (Guo et al., 2019). Incubation with these cells and subsequent cell fractionation were performed as described in the main manuscript. U251-Luc cells exhibited significantly greater cell surface binding and cytoplasm + nuclear uptake than U87MG or GL261 cells (P<0.0001; **Fig. S6**). There were no significant differences observed in binding and internalization into GL261 cells compared to U87MG cells. These results indicate that high EGFR expression is required for effective binding and internalization of panitumumab-AuNPs into GBM cells.

**
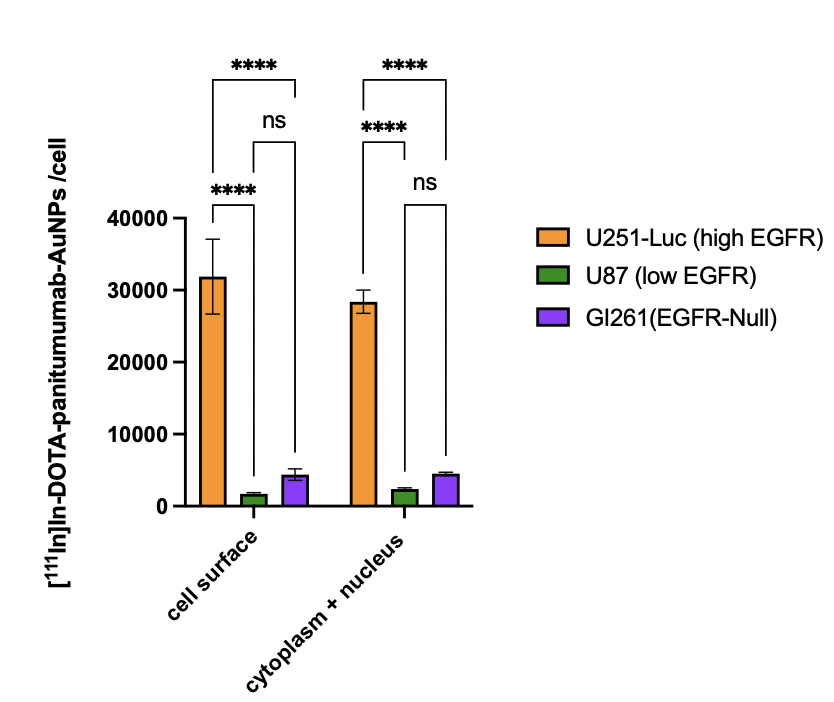
**

**Fig. S6.** Activity on the cell surface or internalized into the cytoplasm + nucleus after incubation of [^111^In]In-DOTA-panitumumab-AuNPs with GBM cells with different EGFR expression levels.

**Cellular dosimetry estimates**

The absorbed doses in the nucleus of U251-Luc cells incubated with panitumumab-[^197^Hg]Hg-AuNPs or non-targeted [^197^Hg]Hg-AuNPs (0.26 MBq; 1 × 10^11^ AuNPs) for 24 h were estimated by cellular dosimetry using previously reported methods (Cai et al., 2023; Cai et al., 2017). Monte Carlo N-Particle (MCNP), code version 6.1 (Los Alamos National Laboratory, Los Alamos, NM, USA) was used to calculate the medium (M) and monolayer S-values (Gy Bq^−1^ s ^−1^ ) (Cai et al., 2017) using cell dimensions [radius of cell (R_C_) = 11.8 ± 0.5 μm and radius of nucleus (R_N_) = 8.3 ± 0.2 μm) measured by confocal microscopy imaging of U251-Luc cells by staining the cell membrane with wheat germ agglutinin-Alexa Fluor 594 immunoconjugate (yellow) and nucleus (blue) with Hoechst 33342 dye (**Fig. S7**) (Cai et al., 2010) It was assimed that there was 200 μL water in each well of a 24-well plate.


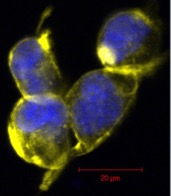


**Fig. S7.** Live cell confocal microscopy images of U251-Luc cells stained with wheat germ agglutinin-Alexa Fluor 594 imunoconjugates to visualize the cell surface (yellow) and Hoechst 33342 dye to visualize the cell nucleus (blue).

The emission spectra for ^197^Hg were obtained from the Medical Internal Radiation Dose (MIRD) decay schemes (Eckerman, 2008). The time integrated activities (Ã in Bq × s) in the medium (M), cell membrane (CM), cytoplasm (Cy), or nucleus (N) were calculated by assuming linear uptake of ^197^Hg in each cell compartment from 0 to 19 h post-incubation, then remaining constant until 24 h when the activity incubated with the cells was removed and the cells seeded for clonogenic survival assay (see main manuscript). The doses in the nucleus (target) from four sources (M, CM, Cy, or N) were calculated as D = ∑Ã_S_ × S_N←S_.

**Table S1 and** **S2** show the self-dose and cross-dose S-values from ^197^Hg homogeneously distributed in the sources CM, Cy and N subcellular compartments of U251-Luc cells or in 200 μL water in each well of a 24-well plate, and the time-integrated activities and the estimated absorbed doses (Gy) in the nucleus of U251-Luc cells incubated with panitumumab-[^197^Hg]Hg-AuNPs or non-targeted [^197^Hg]Hg-AuNPs (0.26 MBq, 1 × 10^11^ AuNPs) for 24 h prior to seeding into 6-well plates for the clonogenic survival assay. Dosimetry calculations assumed that all activity was ^197g^Hg (although ^197m^Hg is co-produced with ^197g^Hg; **Fig. S2**), since at the time of synthesis of [^197^Hg]Hg-AuNPs (~48 h post-production) >90% was ^197g^Hg, due to its 2.7-fold longer half-life. The percentage of ^197g^Hg continues to increase with time. However, the small percentage of ^197m^Hg activity (<10%) slightly overestimates the ^197g^Hg activity measured in the dose calibrator, and further overestimates the calculated absorbed dose by <15%, assuming 100% ^197g^Hg contribution. On the other hand, the ^197^Hg emitted radiation may interact with the AuNPs, attenuating the energy absorbed in the nucleus of GBM cells or in a tumour. This effect wa not considered and may slghtly underestimate the doses. We are currently conducting dosimetry modeling to assess this effect.

**Table S1** S-values and time-integrated activity of ^197^Hg in the cell membrane, cytoplasm and nucleus for U251/Luc cells treated with panitumumab-[^197^Hg]Hg-AuNPs or non-targeted [^197^Hg]Hg-AuNPs for 24 hours in clonogenic survival assay ^a^

| **Source ^c^** | **S value (GyBq^-1^s^-1^)** | | **Ã (Bq.s) ^b^** | |
| --- | --- | --- | --- | --- |
|  | **Self ^d^** | **Cross ^e^** | **Panitumumab-[^197^Hg]Hg-AuNPs** | **[^197^Hg]Hg-AuNPs** |
| CM | 0.000065 | 0.00019 | 3759 ± 1701 | 202 ± 44 |
| Cy | 0.000117 | 0.00019 | 4337 ± 835 | 360 ± 74 |
| N | 0.001152 | 0.00019 | 4775 ± 2040 | 256 ± 65 |
| M | 3.03 × 10^-12^ | | 2.00 × 10^10^ | 2.00 × 10^10^ |

^a^ Cells were treated with 0.26 MBq (1 × 10^11^ AuNPs) for 24 h

^b^ Time-integrated activity in the source compartment from 0 h to 24 h

^c^ Source compartment: CM (cell membrane), Cy (cytoplasm), N (nucleus), M (medium)

^d^ Self-dose S-factor, i.e. sources CM, Cy and N and target N are from the same cell

^e^ Cross-dose S-factor, i.e. sources CM, Cy and N are from surrounding cells of the cell with the targeted N

**Table S2** Estimated absorbed doses (Gy) in the nucleus of U251-Luc cells treated with panitumumab-[^197^Hg]Hg-AuNPs or non-targeted [^197^Hg]Hg-AuNPs in clonogenic survival assay ^a^

| **Source ^b^** | **Panitumumab-[^197^Hg]Hg-AuNPs** | | **[^197^Hg]Hg-AuNPs** | |
| --- | --- | --- | --- | --- |
|  | **Self ^b^** | **Cross ^c^** | **Self ^b^** | **Cross ^c^** |
| CM | 0.24 ± 0.11 | 0.71 ± 0.32 | 0.013 ± 0.003 | 0.038 ± 0.008 |
| Cy | 0.51 ± 0.10 | 0.82 ± 0.16 | 0.042 ± 0.009 | 0.069 ± 0.014 |
| N | 5.50 ± 2.35 | 0.91 ±0.39 | 0.30 ± 0.08 | 0.049 ±0.012 |
| CM+Cy+N | 6.3 ± 2.4 | 2.4 ± 0.6 | 0.35 ± 0.08 | 0.16 ±0.02 |
| M | 0.06 | | 0.06 | |
| All | 8.8 ± 2.9 | | 0.57 ± 0.2 | |

^a^ Cells were treated with 0.26 MBq (1 × 10^11^ AuNPs) for 24 h

^b^ Source compartment: CM (cell membrane), Cy (cytoplasm), N (nucleus), M (medium)

^c^ Self-dose, i.e. sources CM, Cy and N and target N are from the same cell

^d^ Cross-dose, i.e. sources CM, Cy and N are from the surrounding cells of the cell with the targeted N

**SPECT/CT Images**

**Fig. S8.** SPECT/CT images at 168 h in two additional mice infused by CED with panitumumab-[^197^Hg]Hg-AuNPs and in two additional mice infused with non-targeted [^197^Hg]Hg-AuNPs.

**Excised Brains**

**Fig. S9.** Excised brains from NRG mice administered panitumumab-[^197^Hg]Hg-AuNPs or non-targeted [^197^Hg]Hg-AuNPs. The presence of AuNPs is indicated by the darker colour of the brain (areas enclosed by the white dotted lines). There appeared to be greater local diffusion of non-targeted than EGFR-targeted panitumumab-conjugated AuNPs.

**References**

Cai Z, Al-Saden N, Georgiou CJ, Reilly RM. Cellular dosimetry of ^197^Hg, ^197m^Hg and ^111^In: comparison of dose deposition and identification of the cell and nuclear membrane as important targets. Int J Radiat Biol 2023;99:53-63.

Cai Z, Kwon YL, Reilly RM. Monte Carlo N-Particle (MCNP) modeling of the cellular dosimetry of ^64^Cu: Comparison with MIRDcell S values and implications for studies of its cytotoxic effects. J Nucl Med 2017;58:339-345.

Cai Z, Pignol JP, Chan C, Reilly RM. Cellular dosimetry of ^111^In using monte carlo N-particle computer code: comparison with analytic methods and correlation with in vitro cytotoxicity. J Nucl Med 2010;51:462-470.

Chen S, Bas M, Happel S, Randhawa P, McNeil S, Kurakina E, Zeisler S, et al. Determination of distribution coefficients of mercury and gold on selected extraction chromatographic resins - towards an improved separation method of mercury-197 from proton-irradiated gold targets. J Chromatogr A 2023;1688:463717.

Eckerman, K.F.E., A., 2008. MIRD Radionuclide Data and Decay Schemes, 2nd edition ed. Society of Nuclear Medicine, Reston, VA, USA.

Facca VJ, Cai Z, Gopal NEK, Reilly RM. Panitumumab-DOTA-^111^In: An epidermal growth factor receptor targeted theranostic for SPECT/CT imaging and Meitner-Auger electron radioimmunotherapy of triple-negative breast cancer. Mol Pharm 2022;19:3652-3663.

Guo G, Gong K, Puliyappadamba VT, Panchani N, Pan E, Mukherjee B, Damanwalla Z, et al. Efficacy of EGFR plus TNF inhibition in a preclinical model of temozolomide-resistant glioblastoma. Neuro Oncol 2019;21:1529-1539.

He YQ, Liu SP, Kong L, Liu ZF. A study on the sizes and concentrations of gold nanoparticles by spectra of absorption, resonance Rayleigh scattering and resonance non-linear scattering. Spectrochim Acta A Mol Biomol Spectrosc 2005;61:2861-2866.

Ku A, Facca VJ, Cai Z, Reilly RM. Auger electrons for cancer therapy - a review. EJNMMI Radiopharm Chem 2019;4:27.

Randhawa P, Olson AP, Chen S. Gower-Fry KL, Hoehr C, Engle JW, Ramogida C., Radchenko V. Meitner-Auger electron emitters for targeted radionuclide therapy: mercury-197m/g and antimony-119. Curr Radiopharm. 2021;14,:394-419.

Reilly RM, Kiarash R, Sandhu J, Lee YW, Cameron RG, Hendler A, Vallis K, Gariepy J. A comparison of EGF and MAb 528 labeled with ^111^In for imaging human breast cancer. J Nucl Med 2000;41:903-911.
